# Application of quantitative second-line drug susceptibility testing at a multidrug-resistant tuberculosis hospital in Tanzania

**DOI:** 10.1186/1471-2334-13-432

**Published:** 2013-09-14

**Authors:** Stellah G Mpagama, Eric R Houpt, Suzanne Stroup, Happiness Kumburu, Jean Gratz, Gibson S Kibiki, Scott K Heysell

**Affiliations:** 1 Kibong’oto National Tuberculosis Hospital, SanyaJuu- Siha, PO Box 12, Kilimanjaro, Tanzania; 2 Kilimanjaro Christian Medical Centre, Kilimanjaro Clinical Research Institute, PO Box 2236, Moshi, Tanzania; 3 Division of Infectious Diseases and International Health, University of Virginia, PO Box 801340, Charlottesville, Virginia 22908, USA

**Keywords:** Multidrug-resistant tuberculosis, Minimum inhibitory concentration, Aminoglycosides, Flouroquinolones, Para-aminosalicylic acid, Ethionamide, *rpoB*, *inhA*, *embB*, *pncA*

## Abstract

**Background:**

Lack of rapid and reliable susceptibility testing for second-line drugs used in the treatment of multidrug-resistant tuberculosis (MDR-TB) may limit treatment success.

**Methods:**

*Mycobacterium tuberculosis* isolates from patients referred to Kibong’oto National TB Hospital in Tanzania for second-line TB treatment underwent confirmatory speciation and susceptibility testing. Minimum inhibitory concentration (MIC) testing on MYCOTB Sensititre plates was performed for all drugs available in the second-line formulary. We chose to categorize isolates as borderline susceptible if the MIC was at or one dilution lower than the resistance breakpoint. *M. tuberculosis* DNA was sequenced for resistance mutations in *rpoB* (rifampin), *inhA* (isoniazid, ethionamide), *katG* (isoniazid), *embB* (ethambutol), *gyrA* (fluoroquinolones), *rrs* (amikacin, kanamycin, capreomycin), *eis* (kanamycin) and *pncA* (pyrazinamide).

**Results:**

Of 22 isolates from patients referred for second-line TB treatment, 13 (59%) were MDR-TB and the remainder had other resistance patterns. MIC testing identified 3 (14%) isolates resistant to ethionamide and another 8 (36%) with borderline susceptibility. No isolate had ofloxacin resistance, but 10 (45%) were borderline susceptible. Amikacin was fully susceptible in 15 (68%) compared to only 11 (50%) for kanamycin. Resistance mutations were absent in *gyrA*, *rrs* or *eis* for all 13 isolates available for sequencing, but *pncA* mutation resultant in amino acid change or stop codon was present in 6 (46%). Ten (77%) of MDR-TB patients had at least one medication that could have logically been modified based on these results (median 2; maximum 4). The most common modifications were a change from ethioniamide to para-aminosalicylic acid, and the use of higher dose levofloxacin.

**Conclusions:**

In Tanzania, quantitative second-line susceptibility testing could inform and alter MDR-TB management independent of drug-resistance mutations. Further operational studies are warranted.

## Background

Management of multidrug-resistant tuberculosis (MDR-TB) poses a major challenge, since resistance to isoniazid and rifampin precludes the use of two key drugs in the anti-TB regimen. Medications used in the treatment of MDR-TB are deemed second-line because of reduced potency, a worse side effect profile and impaired treatment efficacy compared to medication in the standard first-line regimen for drug-susceptible TB (isoniazid, rifampin, ethambutol, pyrazinamide). Consequently, current World Health Organization guidelines recommend at least 20 months of treatment duration with five drugs (a second-line injectable agent given for at least 8 months: capreomycin, kanamycin, or amikacin; a fluoroquinolone; ethionamide or prothionamide; pyrazinamide; and cycloserine or para-aminosalicylic acid) [1,2]. Yet the guidelines expose the paucity of quality evidence with which to generate treatment recommendations, particularly with regard to the optimal composition and duration of MDR-TB regimens [3].

The lack of rapid and reliable susceptibility testing for the drugs which compose the MDR-TB regimen further limits the ability to assign the ideal combination to an individual patient. Despite individualized treatment existing as the standard of care in areas with adequate expertise and laboratory capacity [4], the lack of second-line susceptibility testing is common in resource-limited settings where the vast majority of MDR-TB occurs, and in such locations empiric regimens have been advocated [1]. Empiric regimens, based on periodic surveillance of drug resistance data, risk inclusion of medications to which the subject’s *Mycobacterium tuberculosis* isolate may be frankly resistant and discounts the pharmacokinetic variability whereby poor circulating drug concentrations impair killing of isolates with borderline susceptibility and which may amplify resistance [5]. Indeed, the gold standard of second-line susceptibility on solid agar uses the proportion method and a single critical concentration of drug to determine qualitative susceptibility or resistance, contrary to the quantitative readout used in the testing of many other infectious pathogens. Furthermore, cross-resistance is not universal among the drugs within the flouroquinolone class or among the injectable agents [6]. As proof of concept, meta-analysis found improved cure rates with use of a later generation fluoroquinolone among patients with extensively drug-resistant isolates resistant to ofloxacin [7].

In Kibong’oto National Tuberculosis Hospital (KNTH) in Tanzania, the country’s only MDR-TB treatment facility, second-line drug regimens are largely empiric as susceptibility testing is available only to isoniazid, rifampin, streptomycin and ethambutol and second-line drugs are procured within a restricted formulary [8]. Given our local knowledge of the gaps in second-line susceptibility testing, we chose to perform quantitative testing by minimum inhibitory concentration (MIC) on a series of isolates from consecutive patients referred to KNTH. MIC testing is now commercially available in a microplate format for the testing of *M. tuberculosis* susceptibility with accuracy of >94% for all first and many second-line drugs, and is now used as the only phenotypic susceptibility testing in large TB public health laboratories [9-11]. In the following study we describe the potential application of quantitative susceptibility testing for drug-resistant TB management in Tanzania.

## Methods

### Study site

KNTH was a former sanatorium and is located in the Kilimanjaro region/Siha district of northern Tanzania ap-proximately 600 kilometers from Dar es Salaam. As the only approved facility for use of second-line anti-TB drugs since November 2009, KNTH received patients from the entire country with MDR-TB, poly-resistant TB (resistant to more than one first-line drug but retaining either isoniazid or rifampin susceptibility) or intolerance to first-line drugs. MDR-TB patients were treated with a standardized regimen during the inpatient intensive phase with one injectable agent (kanamycin, amikacin or capreomycin pending stock availability), a fluoroquinolone (levofloxacin or ofloxacin), ethambutol (if susceptible), pyrazinamide, ethionamide and cycloserine (or para-aminosalicylic acid). Monthly sputum cultures were obtained in pulmonary TB and monitored for conversion to negative per hospital protocol.

### Laboratory methods

All subjects were initially referred to KNTH with first-line susceptibility testing by agar proportion at the national reference laboratory in Dar es Salaam. Use of the rapid molecular diagnostics, GenoType MTBDRplus (Hain Lifescience, Nehren, Germany) and GeneXpert MTB/RIF assay (Cepheid, CA, USA) also contributed to referral in more recent cases. In addition, KNTH retained affiliation with the biotechnology laboratory of the Kilimanjaro Clinical Research Institute (KCRI) which in addition to DNA probe for *M. tuberculosis* complex (Gen-Probe, San Diego, USA) and mycobacterial culture and conventional first-line susceptibility testing by Bactec MGIT (BD, Franklin Lakes, USA), performed MIC testing on MYCOTB Sensititre plates (TREK Diagnostics, Cleveland, USA).

MIC testing was completed as previously described [9,10] on a batch of consecutive *M. tuberculosis* isolates and not available for therapeutic decisions. Prior to the study MIC plates were performed in duplicate with excellent agreement and rare discrepancies within one dilution. Thus for the study, single MIC plates were read manually by inverted mirror at 14 and 21 days by two independent technicians. Resistance breakpoints were based on prior published studies with MYCOTB Sensititre plates and from critical concentrations in similar Middlebrook 7H9 liquid media [9-12], as follows: isoniazid MIC >0.25 μg/ml (pre-filled wells of 0.06, 0.12, 0.25, 0.5, 1.0, 2.0 and 4.0 μg/ml) [9,10]; rifampin >1.0 μg/ml (pre-filled wells of 0.12, 0.25, 0.5, 1.0, 2.0, 4.0, 8.0 and 16.0 μg/ml) [9,10,12], rifabutin >0.5 μg/ml (pre-filled wells of 0.12, 0.25, 0.5, 1.0, 2.0, 4.0, 8.0 and 16.0 μg/ml) [9,10,13], ethambutol >4.0 μg/ml (pre-filled wells of 0.5, 1.0, 2.0, 4.0, 8.0, 16.0 and 32.0 μg/ml) [9,10,12], streptomycin >2.0 μg/ml (pre-filled wells of 0.25, 0.5, 1.0, 2.0, 4.0, 8.0, 16.0 and 32.0 μg/ml) [9,10], kanamycin >5.0 μg/ml (pre-filled wells of 0.6, 1.2, 2.5, 5.0, 10.0, 20.0, and 40.0 μg/ml) [9,10], amikacin >1.0 μg/ml (pre-filled wells of 0.12, 0.25, 0.5, 1.0, 2.0, 4.0, 8.0 and 16.0 μg/ml) [12,13], ofloxacin >2.0 μg/ml (pre-filled wells of 0.25, 0.5, 1.0, 2.0, 4.0, 8.0, 16.0 and 32.0 μg/ml) [9,10,12], moxifloxacin >0.25 μg/ml (pre-filled wells of 0.06, 0.12, 0.5, 1.0, 2.0, 4.0 and 8.0 μg/ml) [12], ethionamide >5.0 μg/ml (pre-filled wells of 0.3, 0.6, 1.2, 2.5, 5.0, 10.0, 20.0 and 40.0 μg/ml) [9,10,12], cycloserine >32.0 μg/ml (pre-filled wells of 2.0, 4.0, 8.0, 16.0, 32.0, 64.0, 128.0 and 256.0 μg/ml) [9,10], para-aminosalicylic acid >2.0 μg/ml (pre-filled wells of 0.5, 1.0, 2.0, 4.0, 8.0, 16.0, 32.0 and 64.0 μg/ml) [9,10,12]. While MIC ranges on solid agar of ‘moderately susceptible’ or ‘moderately resistant’ have been applied for clinical interpretation at specialized centers [14,15], we chose to categorize MICs at or one dilution lower than the concentration for the resistance breakpoint as borderline susceptible. The term ‘intermediate’ susceptibility was avoided given its use in other bacterial infections for which standard breakpoints are set based on large-scale consensus [16]. Quantitative susceptibility testing for pyrazinamide was not available.

For additional comparison to quantitative susceptibility, DNA was amplified and sequenced for known drug-resistance determining regions of *katG* (high level isoniazid resistance), *inhA* (ethionamide and low level isoniazid resistance), *rpoB* (rifampin), *embB* (ethambutol), *gyrA* (fluoroquinolones), *rrs* (amikacin, kanamycin, capreomycin), and *eis* (kanamycin) using methods described from the US Centers for Disease Control [17]. For *pncA* (pyrazinamide), the entire open reading frame and upstream promoter region were amplified. Comparison was made with published sequences for *M. tuberculosis* H37Rv using GeneDoc 2.7.0.

### Statistical analysis

Basic demographic data were abstracted from patient charts along with clinical information including HIV status, prior TB treatment episodes, duration of sputum culture conversion in months (if applicable) and drugs in the treatment regimen during the inpatient intensive phase. Data were entered into Microsoft Excel (Version 14.1.3) and analyzed using SPSS (Version 19).

The proportion of susceptible, borderline susceptible and resistant was reported as simple frequencies for each drug included on the MYCOTB Sensitire plate. Medians and range were used to describe the number of drugs within the study population for which a medication modification was probable. A probable medication modification was defined as a borderline susceptible or resistant drug in the patient’s regimen for which there was an alternative susceptible drug available, or for a borderline susceptible isolate, a dose increase was possible [4]. Medication modification was restricted to second-line drugs for which Bactec MGIT susceptibility was not available. The number of drugs for which a medication modification was probable and the duration of sputum culture conversion were compared by chi-square with p-value of less than 0.05 considered significant.

Ethical approval was obtained from the institutional review boards of the Kilimanjaro Christian Medical University and the University of Virginia, and KNTH hospital management.

## Results

Isolates from 22 patients were available for analysis. Sixteen patients (73%) were male with a mean age of 39 years ± 14. Five (23%) were HIV infected with a mean CD4 count of 342 cells/μl (minimum 242- maximum 443). All patients had at least one prior episode of TB and 9 (41%) had three or more prior episodes. All patients had pulmonary TB and the median duration of culture conversion was 2 months (IQR 1–3). Time to culture conversion did not vary by HIV status.

All 22 isolates were confirmed as *M. tuberculosis* complex by probe. Thirteen (59%) were MDR following repeat susceptibility testing in Bactec MGIT, while the remainder had other resistance patterns to the first-line drugs. For the first-line drugs, MIC testing did not differ in agreement between two technicians and was compared to the conventional Bactec MGIT result (Table 1). For isoniazid, only one isolate was discrepant with a MIC > 4.0 μg/ml but susceptible in Bactec MGIT. DNA was available for sequencing and found to be wildtype for both *inhA* and *katG*. Similarly for rifampin, 20 (91%) correlated, but one isolate, read as susceptible for rifampin by Bactec MGIT, had a MIC indicative of resistance (>16 μg/ml). *rpoB* was wild-type by both sequencing and GeneXpert MTB/RIF assay, supportive of the Bactec MGIT result. Interestingly, the one isolate susceptible by MIC testing that was resistant to rifampin in Bactec MGIT had a MIC of 1.0 μg/ml at the resistance breakpoint. Accuracy of ethambutol was only 59%, due largely to Bactec MGIT susceptible isolates with a MIC one dilution above the resistance breakpoint.

**Table 1:** Minimum inhibitory concentration (MIC) for first-line drugs compared to Bactec MGIT results (N = 22 isolates)

**Drug and MIC result**	**MGIT susceptible**	**MGIT resistant**	**Accuracy**
Isoniazid			95.5%
MIC Susceptible	4	0	
MIC Resistant	1	17^a^	
Rifampin			90.9%
MIC Susceptible	8	1^b^	
MIC Resistant	1	12^c^	
Streptomycin			90.9%
MIC Susceptible	13	0	
MIC Resistant	2	7	
Ethambutol			59.1%
MIC Susceptible	11	1^e^	
MIC Resistant	7^f^	2	

^a^All with isoniazid MIC ≥ 4 μg/ml. ^b^Rifampin MIC of 1 μg/ml. ^c^All with rifampin MIC >16 μg/ml. ^e^Ethambutol MIC of 4 μg/ml. ^f^Five (71%) with ethambutol MIC of 8 μg/ml.

For the second-line drugs tested, a range of MICs were observed (Table 2). For example, while only 1 isolate (5%) was resistant to kanamycin, 10 (45%) were of borderline susceptibility. Similarly, no isolates were ofloxacin resistant, but 10 (45%) were of borderline susceptibility. In contrast, 3 (14%) of isolates were ethionamide resistant and another 8 (36%) were of borderline susceptibility, while para-aminosalicylic acid was fully susceptible in 15 (68%) of cases.

**Table 2:** Minimum inhibitory concentration (MIC) for second-line drugs (N = 22 isolates)

**Drug**	**Susceptible (% N)**	**Borderline susceptible (% N)**	**Resistant (% N)**
	**[MIC range]**	**[MIC range]**	**[MIC range]**
Kanamycin	11 (50)	10 (45)	1 (5)
	[≤0.6-1.2 μg/ml]	[2.5-5.0 μg/ml]	[>40.0 μg/ml]
Amikacin	15 (68)	6 (27)	1 (5)
	[≤0.12-0.25 μg/ml]	[≤0.5-1.0 μg/ml]	[16 μg/ml]
Ofloxacin^a^	12 (55)	10 (45)	0
	[≤0.25-0.5 μg/ml]	[1.0-2.0 μg/ml]	N/A
Moxifloxacin	11 (50)	10 (45)	1 (5)
	[≤0.6 μg/ml]	[≤0.12-0.25 μg/ml]	[0.5 μg/ml]
Ethionamide	11 (50)	8 (36)	3 (14)
	[0.5-1.2 μg/ml]	[2.5-5.0 μg/ml]	[10.0-40.0 μg/ml]
Cycloserine	17 (77)	5 (23)	0
	[1.2-8.0 μg/ml]	[16.0-32.0 μg/ml]	N/A
PAS	15 (68)	2 (9)	5 (23)
	[≤0.5 μg/ml]	[1.0 μg/ml]	[8.0- >64.0 μg/ml]
Rifabutin	9 (41)	0	13 (59)
	[≤0.12 μg/ml]	N/A	[1.0- >16.0 μg/ml]

*PAS* para-aminosalicylic acid. ^a^Patients received ofloxacin or levofloxacin. Moxifloxacin and rifabutin were not available on formularly.

DNA was extracted and amplified from 13 isolates with MIC testing, including 8 (62%) with MDR-TB. For isoniazid, *inhA* was wildtype in all isolates, and *katG* mutated in all resistant isolates when comparing to Bactec MGIT susceptibility (Table 3). Complete agreement was also found for *rpoB* mutation and rifampin resistance when comparing to the Bactec MGIT result. Yet for ethambutol, only one isolate was resistant in Bactec MGIT but lacked *embB* mutation, whereas in the 12 remaining isolates susceptible to ethambutol, 6 (50%) had *embB* mutation. In contrast, by MIC testing for ethambutol, 3 isolates were found resistant and 2 (67%) had an *embB* mutation while 7 isolates were of borderline susceptibility and 3 (43%) had *embB* mutation. No isolate had *rrs* or *eis* mutation despite one isolate with MICs to kanamycin of >40 μg/ml and amikacin of 16 μg/ml, both in the resistant range. Eleven (85%) had mutation in *gyrA* but at a site known to be phenotypically silent and not conferring of resistance (Ser95Thr) [18] and correspondingly none of the 13 isolates had an ofloxacin MIC >2.0 μg/ml. However, 10 (77%) had point mutations in *pncA*, including 6 (47%) conferring amino acid changes or stop codons.

**Table 3:** Molecular targets of resistance distributed by Bactec MGIT result or category of minimum inhibitory concentration (MIC) (N = 13 isolates)

**Drug; gene**	**MGIT**	**MGIT**	**MIC**	**MIC**	**MIC**
	**Susceptible**	**Resistant**	**Susceptible**	**Borderline**	**Resistant**
				**susceptible**	
Rifampin; *rpoB*					
wildtype	5	0	4	N/A	1
mutation	0	8	0	N/A	8
Isoniazid; *inhA* or *katG*					
wildtype	4	0	2	N/A	1
mutation	0	9	0	N/A	10
Ethambutol; *embB*					
wildtype	6	1	2	4	1
mutation	6	0	1	3	2
Ofloxacin; *gyrA*					
wildtype/silent mutation	N/A	N/A	6	7	N/A
mutation	N/A	N/A	0	0	N/A
Amikacin; *rrs* or *eis*					
wildtype	N/A	N/A	8	4	1
mutation	N/A	N/A	0	0	0
Ethionamide; *inhA*					
wildtype	N/A	N/A	5	7	1
mutation	N/A	N/A	0	0	0

*embB* mutation included: Glu378Ala in 2 isolates; Met306Val, Met306Ile, Gly406Asp, Gly406Cys, Tyr319Cys in single isolates. Pyrazinamide susceptibility was not performed but *pncA* mutations included: Val128Gly in 2 isolates; Glu111stop, Asp49Gly, Ser179Ile, and Val169Ala in single isolates. Val128Gly and Val169Ala have not previously been reported. Five isolates (63%) resistant to both isoniazid and rifampin had *pncA* mutation.

The pattern of second-line non-susceptibility was difficult to predict and led to a highly variable number and class of medications where modification was probable. Of the MDR-TB patients, 10 (77%) had at least one medication with probable modification (median 2, maximum 4) (Table 4). The most common medication modification was changing ethionamide to para-aminosalicylic acid, which was probable in 7 (54%). Additionally, there were 3 patients (23%) on kanamycin with borderline or resistant MICs while amikacin retained full susceptibility. Among the non-MDR patients, all 9 (100%) had at least one medication with probable modification.

**Table 4:** Distribution of probable medication changes for MDR-TB patients (N = 13)

**Modification**	**Frequency (% N)**
Ethionamide *change* to para-aminosalicylic acid	7 (54)
Ofloxacin or levofloxacin *change* to high-dose levofloxacin	6 (46)
Kanamycin *change* to amikacin	3 (23)
Amikacin or kanamycin *empiric change* to capreomycin^a^	3 (23)
Amikacin *change* to kanamycin	1 (8)

^a^Term *empiric change* used because amikacin and kanamycin were both resistant or of borderline susceptibility, and use of capreomycin has been empirically advocated in this setting [1], but capreomycin testing was not performed in this study. In 2 subjects with borderline susceptible cycloserine another class switch was not available. They were each receiving cycloserine at 500 mg daily and while escalation of dose to 750 mg daily is possible (split in two doses in morning and before bed), such increase may have prohibitive neuropsychiatric toxicity.

Comparison of culture conversion was restricted to MDR-TB patients only given the more rapid culture conversion expected for patients continued on either isoniazid or rifampin. Among the MDR-TB patients, there were 6 (46%) that converted their sputum culture to negative in ≤ 2 months while the remainder required 3 months or more. In the early culture converters 4 (67%) had ≤1 medication with probable modification compared to 3 (43%) of late culture converters (p = 0.59).

## Discussion

In this study of patients referred to a TB hospital in Tanzania for second-line therapy, the majority had one or more medication that could have rationally been modified with the application of quantitative susceptibility. The frequency of borderline susceptible or resistant medications are of particular importance given that the total number of active medications in a MDR-TB regimen is one of the few factors predictive of treatment success [19]. We harbor further concern for later outcomes such as relapse given that *pncA* mutation was common and the suspected pyrazinamide resistance may negate another key drug in the empiric second-line regimen. While patients with a longer duration of sputum culture conversion had more medications with probable modification this association was not statistically significant given the limited number of subjects with MDR-TB for comparison. Importantly, patterns of non-susceptibility were difficult to predict, further emphasizing the potential utility of individualized testing.

Ethionamide was resistant or borderline susceptible in half of all isolates. Bactericidal at high enough concentrations, ethionamide remains the favored drug among the group IV agents of cycloserine, terizidone and para-aminosalicylic acid, and has been associated with improved cure rates for MDR-TB [2]. Yet ethionamide is a structural anologue of isoniazid and has shown cross resistance [20-22], particularly when there is mutation in the *inhA* promoter region. Our analysis was limited to only one isolate with ethionamide resistance by MIC where DNA sequencing was performed and *inhA* was not mutated. Furthermore, the achievable peak serum concentration of ethionamide (1–5 μg/ml) are very near the critical concentration used to define in vitro resistance and thus isolates of borderline susceptibility may be particularly at risk of inadequate killing in a patient with suboptimal serum drug concentrations [23]. As cycloserine was used in conjunction with ethionamide in the majority of the cases studied, the possible change from ethionamide to para-aminosalicylic acid represented the most likely clinical action among MDR-TB patients.

**Figure 1:**
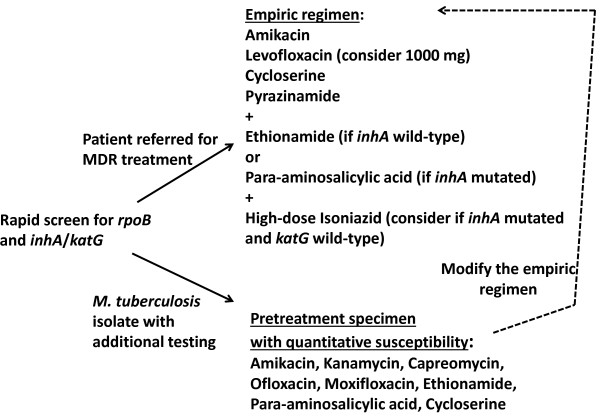
**Potential application of quantitative second-line drug-susceptibility testing for treatment optimization at a multidrug-resistant referral hospital.** Legend. Dotted line represents modification of empiric regimen based on quantitative susceptibility.

Fortunately frank resistance to ofloxacin was absent, but there were many patients with isolates of borderline susceptibility that may have benefited from higher dose levofloxacin or an alternative later generation flouroquinolone [4,24,25]. Reports continue to suggest that later generation fluoroquinolones retain susceptibility against ofloxacin non-susceptible strains [24] and for levofloxacin, the best pharmacokinetic properties appear to be at dose of 1000 mg daily [25,26]. All patients prescribed levofloxacin at KNTH were given 750 mg daily and thus empiric dosing of 1000 mg, except at the lowest patient weight range, is worth consideration (Figure 1). Such dosing may prove particularly relevant for borderline susceptible isolates given recent pharmacokinetic studies suggesting that in patients with MDR-TB and an ofloxacin MIC of 2.0 μg/ml treated with standard doses of ofloxacin, none achieved the target serum area under the time curve (AUC)/MIC [27]. Yet, while the resistance breakpoint for ofloxacin of >2.0 μg/ml appears consistent across platforms of solid and liquid media [12], moxifloxacin has varied from 0.5 μg/ml for the agar proportion method on Middlebrook 7H10 and 7H11, and 0.25 μg/ml in Bactec MGIT which employs an enriched 7H9 liquid media similar to the MYCOTB Sensititre plate. However, recent studies of MYCOTB Sensititre in comparison to agar proportion have suggested a moxifloxacin MIC of >2 μg/ml as resistant [9,10] but there were few isolates resistant on agar and the number with moxifloxacin MIC in the range of 0.25-2.0 μg/ml was not explicitly stated. Of note, all 22 isolates in our study had a moxifloxacin MIC of ≤0.5 μg/ml and in the isolates sequenced, none had a *gyrA* mutation. Such discrepancy highlights the importance of acquiring further quantitative susceptibility in a standardized platform on isolates with well characterized epidemiology.

The application of quantitative susceptibility for optimization of drugs within the MDR-TB regimen may be all the more important given the challenges of routine pyrazinamide susceptibility testing. More than 60% of the MDR isolates available for sequencing in this study had a mutation resultant in amino acid change or a stop codon in the *pncA* gene, including two mutations in three isolates not previously reported. *pncA* encodes the pyrazinamidase responsible for converting pyrazinamide to the active form of pyrazinoic acid and mutations in the gene perform reasonably well for prediction of resistance with sensitivity of 85% and specificity of 86% in a large series of isolates from the US Centers for Disease Control [17]. However genotyping for *pncA* requires substantial amplification and sequencing across the entire 558 base pair gene [28]. Additionally, conventional methods are technically demanding and poorly reproducible given the requirement for acidified media and attendant growth constraints. Thus, pyrazinamide has been excluded from commercial MIC plates and ongoing research will be required to determine if mutation in any specific region of the *pncA* gene will be associated with a more substantial quantitative change in susceptibility than other regions of the gene.

For the remainder of the drugs beside pyrazinamide that make up a typical MDR-TB regimen, we favor the relative ease of the quantitative MIC plate in set up, reading of results, and the fact that it contains quantitative information that is additive to molecular testing alone. As a screening tool however prior to referral for MDR-TB treatment, our results suggest that in Tanzania *inhA* and *katG* genotypic methods could be utilized. Further study is required to determine if *inhA* may be used to determine the empiric choice of ethionamide or para-aminosalicylic acid pending MIC testing (Figure 1). In contrast, given the low proportion of isolates with injectable agent resistance and the lack of *rrs* or *eis* mutation noted in the isolates available for sequencing, as well as the absence of *gyrA* mutation in the isolates of borderline susceptibility to ofloxacin, new line-probe assays for these targets may be of less value [29]. Instead, MIC testing would allow for selection within the class of aminoglycosides and support the use of higher dose levofloxacin (Figure 1).

Of note, there was a seemingly better correlation with *embB* mutation and the MIC plate for ethambutol than to the Bactec MGIT result. This may explain the poor specificity of *embB* observed in field studies using MGIT as the comparator [6]. While such discrepancies can occur in a qualitative susceptibility platform for any isolate with a MIC near the critical concentration, this appears particularly common for ethambutol when using MGIT as the comparator, and laboratories such as the Florida Bureau of Public Health that use the Sensitire MYCOTB plate exclusively for phenotypic susceptibility testing, now report an intermediate range for ethambutol, streptomycin and isoniazid [11]. Furthermore, all *embB* mutations found in this cohort have been associated with in vitro resistance in other studies, but commercial line-probe assays have focused on a single codon (306) of the gene [17,29], further dampening enthusiasm for their utility in our setting.

In summary, we believe quantitative susceptibility methods would prove a worthwhile investment similar to other MDR-TB programs where individualized management based on second-line drug-susceptibility has been modeled as cost-effective [30,31]. For example, despite the subsidized price for second-line medications in Tanzania procured through the WHO Green Light Committee initiative, a switch from kanamycin ($0.53 USD per typical injection) to amikacin ($0.15 USD) [32], as was the potential for 23% of the MDR patients studied based on MIC testing, would pay for the cost of the MIC plate (~$40 each) during the 8 months or more of drug administration. While a major advantage of the commercial plate was the lyophilized drug of each concentration in the prefilled wells, and the ease of implementation into a laboratory already performing TB culture, similar in-house platforms could be developed that may ultimately defray cost.

There are several limitations to our analysis that warrant consideration. The findings were restricted to a small number of clinical isolates and cannot necessarily be generalized to other settings. Additionally, the critical concentrations used to define qualitative resistance for second-line medications remain a subject of debate, are influenced by the platform of testing, and ultimately require rigorous clinical follow-up among large numbers of patients [33]. As such our categorization of borderline susceptibility may be imprecise, particularly for medications such as cycloserine that are not entirely concentration dependent in their activity. Also, our only marker of treatment success was culture conversion, which is also dependent upon numerous host factors. We were unable to determine if patients with borderline susceptible or resistant isolates, particularly those of a certain class of drug, had a higher likelihood of worse late outcomes such as reversion of culture positivity during the outpatient continuation phase, or relapse following treatment completion. Despite these limitations we believe quantitative susceptibility testing holds promise for second-line regimen optimization in Tanzania and further studies to explore the use of such testing should be pursued.

## Conclusions

In Tanzania, quantitative second-line susceptibility testing could inform and alter MDR-TB management independent of drug-resistance mutations. Further operational studies are warranted with a standardized platform for MIC testing and informed by resistance breakpoints from isolates with well characterized epidemiology and clinical outcome.

## Abbreviations

MDR-TB: Multidrug-resistant tuberculosis; MIC: Minimum inhibitory concentration; MGIT: Mycobacteria growth indicator tube.

## Competing interests

The authors declare that they have no competing interests.

## Authors’ contributions

SGM and SKH conceived of study, performed data analysis and drafted the manuscript. SS performed DNA sequencing and provided critical revision of the manuscript. HK and JG performed mycobacterial culture and susceptibility testing, and provided critical revision of the manuscript. ERH and GSK interpreted data and provided critical revision of the manuscript. All authors read and approved the final manuscript.

## Pre-publication history

The pre-publication history for this paper can be accessed here:

http://www.biomedcentral.com/1471-2334/13/432/prepub
